# Influence of polymer additives on the rheological properties of bitumens

**DOI:** 10.1039/d6ra03668f

**Published:** 2026-07-17

**Authors:** Leylufer I. Aliyeva, Sanan S. Goyushlu

**Affiliations:** a Institute of Petrochemical Processes Named after Academician Y.H. Mammadaliyev of the Ministry of Science and Education Khodjaly Ave., 30 AZ 1025 Baku Azerbaijan; b SOCAR Heydar Aliyev Oil Refinery Oktay Valiyev Str. 1 AZ1060 Baku Azerbaijan senangoyuslu002@gmail.com

## Abstract

It is important to incorporate polymers into bituminous binders to improve the quality and performance of asphalt. Currently, polymer-modified asphalt binders (PMBs) play an important role in road engineering. Polymer modifiers absorb the maltene fractions of bitumen and form a three-dimensional network within the binder. The service life of polymer-modified asphalt pavements is approximately 24% longer than that of conventional pavements. Polymer additives are used to improve the properties of bitumen, such as increasing its strength and resistance to loads and extreme temperatures. Common additives include elastomers such as styrene-butadiene-styrene (SBS) and styrene-butadiene rubber (SBR). Plastomers such as ethylene vinyl acetate (EVA) are also widely used. These additives improve the stiffness and high-temperature performance. These modifications create polymer-modified bitumen, which is used in road surfaces to prevent cracking, rutting, and other types of wear, especially in areas with heavy traffic or extreme climates. Some of the main types of polymer additives include SBS, a common elastomer that improves elasticity and flexibility, making asphalt more resistant to cracking and rutting, especially at high temperatures and under heavy loads, and EVA, a plastomer that gives bitumen stiffness and strength. Styrene-butadiene rubber (SBR) is another elastomer used to modify bitumen and exhibits properties similar to SBS. Crumb rubber is a recycled material obtained from waste tires and can be used as an alternative modifier. Other additives include ethylene-propylene copolymers and various polyalkyl methacrylates, although the latter materials sometimes have limited applicability. The advantages of polymer modification include increased durability (makes the asphalt pavement more resistant to wear), increased thermal stability (improves performance at both high temperatures (rutting) and low temperatures (cracking)), increased elasticity and recovery (improves flexibility, allowing the pavement to better withstand loads), and reduced maintenance requirements (extends the service life of the pavement). This paper provides a review of research studies on the use of polymer additives to improve the rheological properties of bitumen. Although numerous studies have investigated individual polymer modifiers to date, there is still no comprehensive comparative review examining the effects of different polymer classes on the rheological behavior, compatibility, and long-term performance of bitumen. Therefore, this review aims to provide a systematic and comparative evaluation of polymer additives used in bitumen modification.

## Abstract 2

It is important to incorporate polymers into bituminous binders to improve the quality and performance of asphalt.

## Graphical abstract

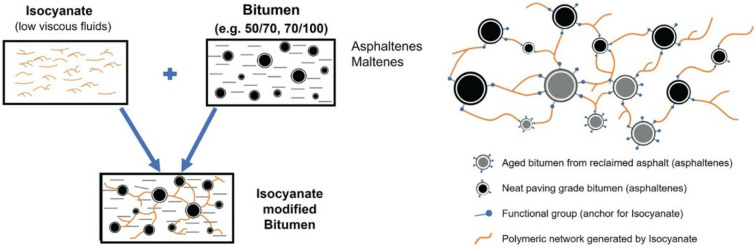

## Introduction

**Fig. 1:**
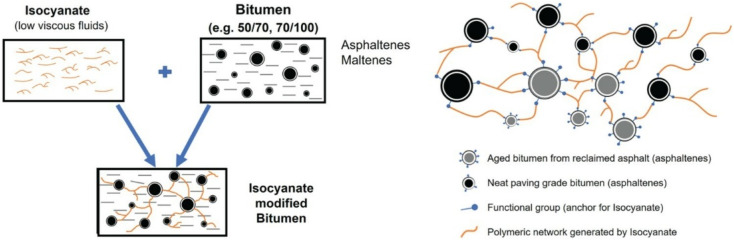
Schematic of the working mechanism of an isocyanate-based additive (BASF, Citation 2020): modification of bitumen with a polyisocyanate-based additive and interactions between a polyisocyanate-based additive and reclaimed asphalt (RA).^14^ Working principle of an isocyanate-based additive. Reproduced from Carreño and Oeser, *Road Mater. Pavement Des.*, 2021, **22**, https://doi.org/10.1080/14680629.2021.1900897, under the terms of the Creative Commons Attribution-NonCommercial-NoDerivatives License (CC BY-NC-ND 4.0).

Petroleum bitumen is the main type of binder used in road and civil construction. Steadily increasing demands for the durability of roads, roofing materials, sealants, and, consequently, the quality of bitumen, as well as increased attention to environmental issues in production, are stimulating the development of new technologies and the improvement of existing production processes. In addition, the addition of polymer modifiers plays a key role in determining the life of the asphalt layer. The better the physical and rheological properties of the bitumen, the longer the life of the asphalt layer. Otherwise, the main difficulties that significantly affect the performance of bitumen are cracking, high-temperature deformation and aging. At low/medium temperatures, the difficulties of thermal cracking and fatigue cracking (horizontal cracks) arise due to the stiff behavior of bitumen due to low-/medium-temperature aging. However, these cracks rapidly reduce the life of the asphalt layer due to water infiltration into the asphalt layer and cause the road surface to fail quickly. Therefore, the bitumen must be hard enough to resist deformation at high temperatures and elastic enough to resist the formation of horizontal cracks at low/medium temperatures. Over the past two decades, the use of styrene-butadiene-styrene (SBS), ethylene vinyl acetate (EVA), crumb rubber (CR), and recycled plastics (PE, PP, and PET) has shown significant improvements in binder elasticity, asphalt composition variation, crack resistance, and environmental sustainability. Modern road materials, particularly polymer-bitumen binders (PBBs) based on styrene-butadiene-styrene (SBS), provide a high level of performance. The rheological properties of bitumen determine its behavior under deformation, including its viscosity, elasticity, plasticity, and crack resistance, which vary depending on the temperature, determining its suitability for road construction (viscoelasticity and extensibility) and waterproofing. The main parameters characterizing rheology are penetration (hardness), softening point, and extensibility, which are standardized for different grades of bitumen (road, insulation, and roofing). The aim of this review is to critically analyze recent studies on the use of polymer additives for improving the rheological properties of bitumen. The performance and viscoelastic properties of both unmodified and modified bitumen were evaluated. The study included performance-based tests, such as frequency sweep and linear amplitude sweep (LAS), to characterize and classify the materials.^1–5^ The bitumen modifications include SBS at concentrations of 4% and 5%, bone powder at concentrations of 4%, 5% and 6%, and waste cooking oil at a concentration of 4–8%. Tests have been conducted on various types of polymer-modified bitumen to see how they maintain their properties in the high temperature range. The introduction of these components into the bitumen formulation significantly increases stiffness, elasticity, and fatigue strength, with SBS-modified samples achieving the highest performance characteristics and fatigue strength. Bitumen modified with waste cooking oil becomes less stiff and has lower fatigue strength, suggesting that waste cooking oil primarily acts as a plasticizer by softening the bitumen. SBS and bone powders are effective modifiers for improving the mechanical and fatigue properties of bitumen and are suitable for applications under challenging conditions. However, although these materials offer environmental benefits, they may reduce structural performance, making them less suitable for heavily loaded pavements. Requirements for the properties of bitumen binders are defined in European standards. The physicochemical properties of bitumen depend on its colloidal structure (asphaltenes dispersed in an oily matrix consisting of saturated, aromatic compounds and resins), which primarily depends on the source of the raw materials and processing. Bitumen properties can be assessed by group composition and elemental analysis, but they are most often characterized using analytical techniques such as DTG, DTA, TGA, ^1^H NMR, and FTIR spectroscopy. Bitumen for road use is assessed based on its physical properties. To improve the quality properties of bitumen and asphalt, additives are used, for example, to increase elasticity, improve thermal stability, enhance adhesion and aging resistance, reduce viscosity, or prevent binder drainage from the aggregate surface.^6–10^ A previous article analyzed the use of three bitumen properties, namely, needle penetration, softening point, and selected additives (Sasobit, Licomont BS100, Wetfix BE and CWM), to improve the adhesion properties of three types of road bitumen, namely, 40/60, 50/70 and polymer-modified 45/80 bitumen. Property measurements were carried out in accordance with the relevant European standards. Laboratory tests showed a significant increase in the softening point of road bitumen grades 50/70 and 35/50 by 13–45 °C. The effect of additives on the softening point of bitumen varies. Penetration varies depending on the types of bitumen and additive used. Penetration values for PMB 45/80–75 bitumen modified with Sasobit and Licomont BS100 additives demonstrate an increase in bitumen hardness by 16 ± 0.1 mm and a change in the particle size distribution. Changes in the penetration and softening point are significantly reflected when calculating the penetration index as a parameter of temperature sensitivity. Additives reduce bitumen viscosity, primarily when using modified bitumen. When the Wetfix BE mix is added to 35/50 grade bitumen, the viscosity increases. The additive alters the properties of the original bitumen binders, which may affect the properties of asphalt concrete mixtures and layers. New regulations governing bitumen vapor and aerosol emissions, as well as increasing traffic and climate loads on highways, require innovative building materials.^11–13^ In a study conducted in Germany, a polymer modifier was added to a 0.5 km asphalt-concrete mixture, and the asphalt retained up to 70% of its original properties at temperatures 15–25 °C lower than the hot mix asphalt (HMA) temperature. In the study, the parameters of the added modifier were measured, and the damage mechanism of the bitumen was studied. In this study, 70/100 grade bitumen was modified with 2.0% by weight of the new isocyanate additive in-line at the asphalt mixing plant without reducing the production time (Fig. 1)^14–19^.

The modifier employed in this study was an isocyanate-based polymer additive. According to the manufacturer's specifications, the material is a black liquid with a density of 1.2 g cm^−3^ and a viscosity of 210 mPa s at 25 °C. These characteristics indicate good fluidity and facilitate its incorporation into the bitumen matrix during the modification process.

Bitumen and asphalt testing were conducted under laboratory conditions on asphalt concrete collected from a construction site. By reducing the temperature, emissions can be significantly reduced while maintaining comparable asphalt performance. Bitumen–clay nanocomposite binders incorporating a styrene-butadiene-styrene (SBS) triblock copolymer, as well as combinations of SBS and crumb rubber (CR) at different CR/SBS ratios, have been synthesized and extensively characterized. In addition to binder formulations, composite samples containing polymer and concrete sand at a weight ratio of 1 : 9 have been prepared and evaluated. The modified binders have been investigated with respect to filler dispersion, storage stability, mechanical performance, and water susceptibility. The results demonstrated that nanoclay-containing systems consistently outperform formulations modified solely with polymer additives. Furthermore, nanocomposite binders incorporating both SBS and CR exhibit the most favorable overall performance, particularly in terms of storage stability. These findings indicate that the partial replacement of CR with SBS, combined with the incorporation of low-cost nanoclay fillers, can produce advanced bituminous materials with enhanced durability, stability, and engineering performance. Bitumen pavements are continuously exposed to heavy traffic loading and adverse environmental conditions, including moisture, temperature fluctuations, and ultraviolet radiation. These factors accelerate binder aging and pavement deterioration, leading to cracking, rutting, and other forms of distress. Consequently, considerable research efforts have focused on modifying bitumen to improve its rheological, mechanical, and durability-related properties (Tables 1 and 2). The incorporation of polymers, crumb rubber, and nanomaterials has emerged as an effective strategy for enhancing pavement performance and extending the service life. Polymer modification is considered one of the most effective approaches for improving bitumen performance, and many modifiers can be obtained from recycled materials rather than virgin chemical products.^20–23^

**Table 1:** Rheological performance comparison of the polymer-modified bitumen systems

Modifier class	Examples of polymers	High-temperature performance (G*/sin(*δ*))	Phase angle (*δ*)	Low-temperature cracking resistance	Main rheological effect
Elastomeric	SBS, SBR, and CR	Very high	More elastic	High resistance	Elastic network formation and fatigue resistance
Plastomer	PE, PP, EVA, and PET	Moderate–high	More viscous	Moderate–low	Stiffening and rutting resistance
Reactive	Isocyanates, epoxies, and EVA-GMA	High (stable)	—	High	Chemical bonding and phase stability
Nano- and bio-based	Nanoclay, PLA, and chitosan	Moderate	Stable	High (aging control)	Interface reinforcement and antiaging

**Table 2:** General characteristics of standard paving bitumen (unmodified).^21^ Reproduced from S. Nizamuddin, Y. J. Boom, F. Giustozzi, *Polymers*, 2021, **13**, 3242, https://doi.org/10.3390/polym13193242, under the terms of the Creative Commons Attribution (CC BY 4.0) license

Property	Value
Density, g/sm^3^	1.004–1.019
Penetration (0.1 mm)	59.10–98.0
Penetration index	0.152–0.601
Softening point, °C	42–65
Flash point, °C	240–350
Fire point, °C	270–376
Ductility (mm)	76–720
Viscosity (cP)	100–465.35
Saturated hydrocarbons, %	4.1–15.9
Aromatic hydrocarbons, %	38.7–69.1
Resin fraction, %	15.1–34.9
Asphaltene fraction, %	9.2–14.2
Colloidal stability index	0.191–0.334

This review article details the use of plastomers as bitumen modifiers, with a special focus on recycled plastics and their potential applications in improving bitumen performance and pavement durability. The review also discusses chemical modifiers that improve the compatibility of plastomers with bitumen. It has been found that plastomers, either alone or in combination with two or three polymers, exhibit high stiffness at high temperatures. Various polymers have been successfully used for bitumen modification, including high-density polyethylene (HDPE), low-density polyethylene (LDPE), linear low-density polyethylene (LLDPE), medium-density polyethylene (MDPE), polypropylene (PP), polystyrene (PS), polyethylene terephthalate (PET), ethylene methyl acrylate (EMA), and ethylene vinyl acetate (EVA). However, each has its own advantages and disadvantages (Table 3), which are discussed in detail in the review. The recent increase in the use of recycled materials in road construction has shed new light on the use of virgin and recycled plastomers for bitumen modification as a low-cost and, in some ways, environmentally beneficial solution in road and pavement construction. The incorporation of these polymers into bitumen significantly enhances its overall performance characteristics. It leads to a marked improvement in penetration resistance, indicating a stiffer and more deformation-resistant binder. At the same time, ductility is increased, which improves the flexibility and cracking resistance of bitumen, especially at low temperatures. The softening point is also significantly elevated, allowing the modified bitumen to perform better under high-temperature conditions, without excessive rutting or flow. In addition, these polymers contribute to improved elasticity, fatigue resistance, and aging performance, making the asphalt more durable under repeated traffic loading and varying climatic conditions.

**Table 3:** Basic characteristics of different plastomers, including PP, EVA, EBA and PE (HDPE, LDPE and L-LDPE).^21^ Reproduced from S. Nizamuddin, Y. J. Boom, F. Giustozzi, *Polymers*, 2021, **13**, 3242, https://doi.org/10.3390/polym13193242, under the terms of the Creative Commons Attribution (CC BY 4.0) license

Property	HDPE	LDPE	L-LDPE	PP	EVA	EBA
Material density, kg m^−3^, ASTM 209B	938–961	890–953	917–944	820–950	920–935	930
Softening point, °C, ASTM D-1525	127	95	110–115	140–150	80–150	130
Tensile strength, MPa	3.1–27	2.34–10.11	13–22	330–414	33	20
Melting point, °C	129–149	106–120	124–128	410–460	290–335	76
Thermal degradation temperature, °C	430–480	406	424–472	410–460	290–335	315
Elongation at break, %, ASTM D 412	500–560	300–700	650	40–350	700–1000	900
Crystallinity, %	52.5–86	35–47.6	48–53	—	40–65	10.6

**Fig. 2:**
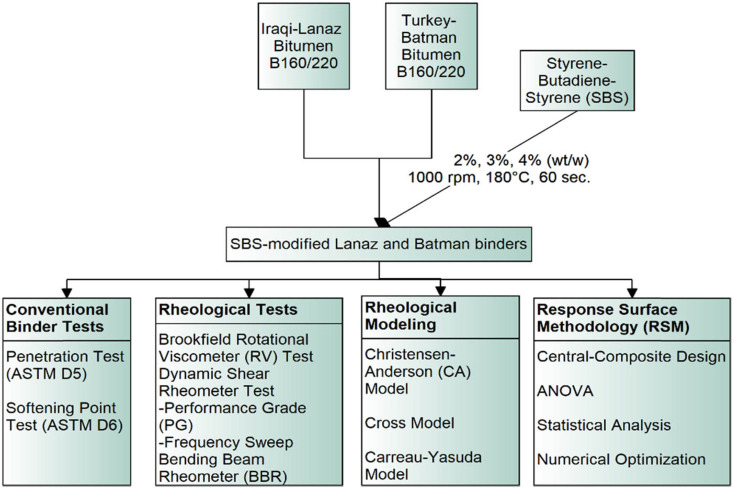
Schematic of the experimental procedure followed in ref. 31. Softening point values of SBS-modified Iraqi bitumen. Reproduced from A. M. Özdemir, B. F. Yalçın, M. Yılmaz, *Appl. Rheol.*, 2025, **35**, https://doi.org/10.1515/arh-2025-0049, licensed under CC BY 4.0.

In order to comprehensively study the diverse and complex effects of polymeric and reactive additives on asphalt binders using experimental studies, a methodology based on traditional binder testing methods (DTA, DTG, HNMR, and IR analyses) was developed.^24–29^ Unlike many previous studies that focused on a limited set of additive types, this review expanded its scope by comparatively analyzing different elastomeric and chemical additives (Table 4), all of which were tested on the same base bitumen using a single standardized procedure (Fig. 2). The evaluations were conducted systematically based on the results of traditional binder tests. The experimental results showed that the Emprenye 611 additive significantly improved high-temperature performance of bitumen, while the CR additive made a noticeable contribution to low-temperature flexibility and crack resistance of bitumen. This comparative methodology sheds light on how different additive types affect the mechanical and rheological properties of asphalt binders. By examining both aspects, this study offers a unique and innovative perspective that can guide the future development of asphalt concrete technology and contribute to the creation of pavements that are more durable under various climatic and loading conditions.

**Table 4:** Properties of the additives used in ref 25. Effects of various reinforcements on the mechanical properties of a plastic block. Compiled by the authors from the published literature

Property	SBS	701	611
Molecular structure	Linear	Linear	Radial
Styrene/butadiene ratio	31/69	33/66	31/69
Density, g cm^−3^	0.95	0.92	0.95
Oil content	—	—	—
Viscosity (5%, cps)	—	14	24
Melt index (190 °C/2.16 kg)	<1	<1	<1
Tensile strength, kgf cm^−2^	325	220	201
Hardness	70	80	84
Ash content	—	0.2	0.2
Elongation at break, %	882	600	71

**Fig. 3:**
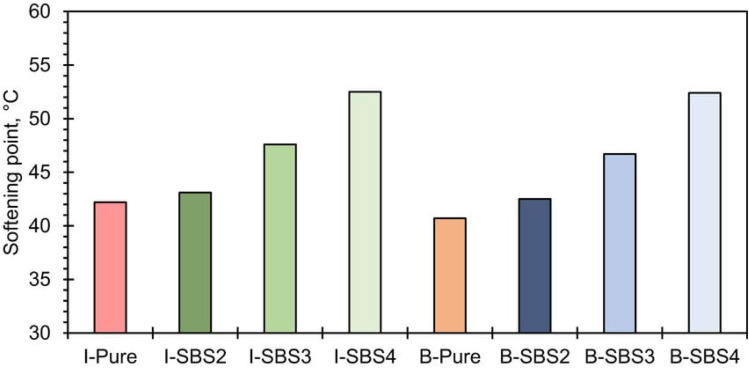
Softening point values of the SBS-modified Iraqi and Batman bitumen.^31^ Softening point values of the SBS-modified Batman bitumen. Reproduced from A. M. Özdemir, B. F. Yalçın; M. Yılmaz, *Appl. Rheol.*, 2025, **35**, https://doi.org/10.1515/arh-2025-0049, licensed under CC BY 4.0.

The table shows that polymer composites incorporated into bitumen significantly modify the rheological and mechanical properties of bitumen depending on the crude oil source. As a result, the composition of bitumen produced from different sources is significantly changed by the polymer composites. In this study, the rheological properties of B 160/220 penetrating binders obtained from the Turkish-Batman and Iraqi-Lanaz refineries modified with the most commonly used polymer modifier styrene-butadiene-styrene (SBS) were compared (Fig. 3).^30–36^

Some additional tests were performed to verify their high-temperature tolerance. In addition to the experimental modifications, the rheological behavior of the binders was analyzed using the Christensen-Anderson, Cross and Carreau-Yasuda models. Time–temperature superposition principle master curves were constructed to characterize their viscoelastic behavior. The results showed that the initial rheological properties of the bitumen, *i.e.*, the composition of the oil from which it was obtained, significantly affect its response to SBS modification. For example, when 4% SBS is added, the G*/sin *δ* value increases by 199% at 64 °C compared with that of the unmodified binders, and the softening point increases by about 4 times, *i.e.*, 9.6 °C. Although the addition of polymer modifiers significantly improves the rheological properties of both types of bitumen, the mechanism of action of the additives varies depending on the source.

Bitumen binders account for 4–7% of the weight of flexible pavements and play a critical role in the structural and functional performance of road surfaces.^37–40^ Traditional hot-mix asphalt (HMA) production is carried out at temperatures of 150–190 °C, resulting in high energy consumption, smoke and odor emissions, and consequently significant cost losses and environmental damage. Growing environmental awareness has called into question the feasibility of HMA production at lower temperatures. Consequently, warm-mix asphalt (WMA) and cold-mix asphalt (CMA) applications were developed as alternatives in a previous study. Softening, flash point, and viscosity were determined based on physical properties. Thermal sensitivity was analyzed, and the rheological properties of the base and short-term aging forms, which require high bitumen processing temperatures, were investigated using a dynamic shear rheometer. Ultimately, the effect of using Sasobit for modification was determined. The study provided a brief assessment of the economic and environmental impacts of using WMA technology. The results showed that the studied bitumen properties, with the exception of the flash point, are significantly improved (Table 5).

**Table 5:** Properties of bitumen used in ref. 38. Reproduced from I. Gökalp, *Eur. J. Tech.*, 2021, **11**(2), 182–189, https://doi.org/10.36222/ejt.966398, under the terms of the Creative Commons Attribution 4.0 International License (CC BY 4.0)

Property	Value
Color	Off-white
Odor	Odorless
Flash point	285.0 °C
Density	0.900 g sm^−3^
Average molecular mass	1000 g mol^−1^
pH	Neutral
Physical form	Solid
Thermal decomposition	250.0 °C
Reaction temperature	>90 °C
Water solubility	Insoluble

Table 6 presents the mixing and compaction temperatures of base bitumen and bitumen modified with different percentages of the Sasobit additive. While the base bitumen required mixing and compaction temperatures of 164.1–161.0 °C and 156.2–151.0 °C, respectively, the addition of 5% Sasobit reduced these temperatures to 156.4–150.7 °C and 142.2–133.6 °C, respectively. This reduction was attributed to the ability of Sasobit to lower the viscosity of bitumen at elevated temperatures, thereby improving its workability. Consequently, asphalt mixtures can be produced and compacted at lower temperatures without compromising the processing efficiency. These findings demonstrate the potential of Sasobit as a warm-mix asphalt additive for reducing energy consumption and environmental emissions during pavement construction.

**Table 6:** Properties of bitumen modified with additives.^38^ Table 6. Reproduced from I. Gökalp, *Eur. J. Tech.*, 2021, **11**(2), 182–189, https://doi.org/10.36222/ejt.966398, under the terms of the Creative Commons Attribution 4.0 International License (CC BY 4.0)

Code	Components	Mixing temperature, °C	Compaction temperature, °C
BB	Base bitumen	164.1–161.0	156.2–151.0
SAS-0.01	BB + 1% Sasobit additive	161.3–157.2	151.0–144.8
SAS-0.02	BB + 2% Sasobit additive	160.0–155.4	148.5–141.5
SAS-0.03	BB + 3% Sasobit additive	159.5–154.6	147.3–139.9
SAS-0.04	BB + 4% Sasobit additive	157.9–152.6	144.8–137.0
SAS-0.05	BB + 5% Sasobit additive	156.4–150.7	142.2–133.6

**Fig. 4:**
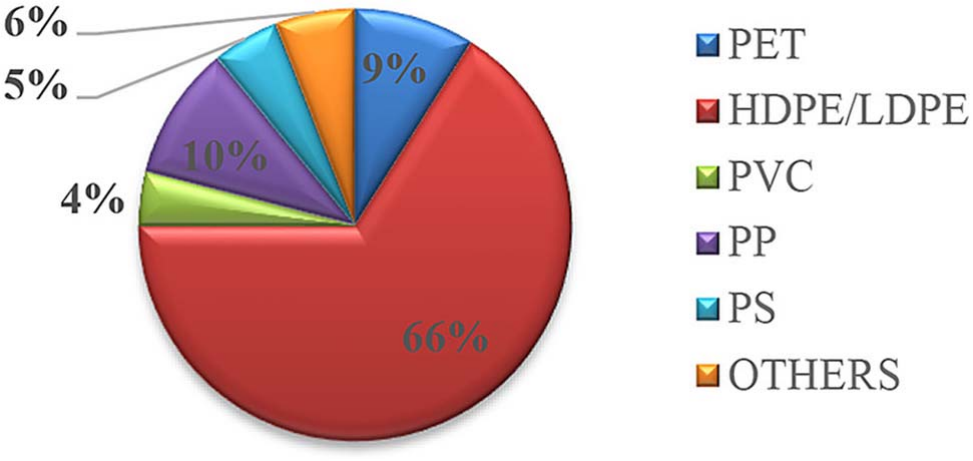
Percentage of polymer waste in India.^55^ Effects of various reinforcements on the mechanical properties of a plastic block. Reproduced from Karthick, K., Ramana, V. M., Muralikrishnan, M. S., Vishnuvardhan, N., & Kumar, S. N., *J. Phys.: Conf. Ser.*, 2021, **2054**, 012075, https://doi.org/10.1088/1742-6596/2054/1/012075, under the terms of the Creative Commons Attribution License (CC BY 3.0).

Various additives, mixtures and modifiers have been used to improve the technical properties and strength of bitumen used in the construction of road surfaces. In some cases, polymer modifier additives are added to bitumen, which is used not only for road surfaces, but also for industrial insulation purposes. As a result, waste materials, complex long-chain polymers, and multicomponent resins or plastics are used as modifiers. For economic and environmental reasons, waste materials are increasingly used as additives, mixtures and modifiers. Currently, the most commonly used physical bitumen modifiers are obtained from copolymers of natural origin or are formed during the synthesis of two substances. Polymers are substances that do not participate in chemical reactions with bitumen, do not act as fillers and do not form a spatial network (also known as physical crosslinking) within bitumen. The developments in organic chemistry have made it possible to synthesize a number of substances that can chemically modify bitumen.^41–47^ The aim of the study in this article is to investigate the suitability and extent of modified natural and post-treatment petroleum waste (diamidoamine dehydrate) as bitumen modifiers. This article investigated the mechanism of the effect of the addition of technical imidazoline on some properties of bitumen. 160/220 bitumen samples, most commonly used for the production of waterproofing products, were analyzed. The following tests were performed on the base bitumen and bitumen modified with technical imidazoline: softening temperature analysis at 25 °C and needle penetration depth analysis. The results showed that the imidazoline modifier used in small amounts significantly improves the thermoplastic properties of bitumen at low temperatures, accelerates the oxidation of bitumen, and prevents the formation of cracks in asphalt. The addition of technical imidazole prevents the early hardening of bitumen, thereby increasing its elasticity and resistance to mechanical damage. Due to the numerous difficulties in the production of polymer bitumen and the search for cheaper, more environmentally friendly solutions, scientists have proposed dozens of polymer modifiers. Imidazolines negatively affect the softening point of bitumen, making their use as a stand-alone modifier impossible. Therefore, the next stage of the authors' research is aimed at creating a hybrid bitumen modifier that combines the positive effects of polymers and imidazoline on the properties of bitumen binders. References 48–53 provide general information on wax additives in bitumen and asphalt-concrete mixtures. These articles focus on asphalt pavements made from polymer-modified bitumen, especially in several European countries. In some cases, wax coatings made from paraffin are added to the asphalt, which significantly improves some of its properties. In addition, brittleness and elongation properties can be improved. These waxes differ significantly from natural bitumen waxes in terms of molecular weight and molecular weight distribution. They deform and melt more quickly than natural wax in bitumen at temperatures above about 100 °C. In some cases, polymer-modified bitumen has poor flowability, but the addition of wax increases the flowability. Scientists and road engineers are considering the mastic asphalt used in Sweden (for bridges, parking lots, *etc.*), as it is more environmentally friendly, easier to handle, and allows various types of modifications. However, paraffin modification should not have a significant negative effect on the performance properties of mastic asphalt. In addition, a hardening effect is observed at medium and high temperatures (below the pavement temperature), which has a positive effect on absolute stability. Regarding low-temperature properties, results show a negative effect on low-temperature cracking susceptibility, and the addition of FT paraffin has a greater effect than the addition of mineral wax. Currently, researchers are studying the use of new nanoscale materials as one of the modifiers of asphalt concrete binders, and work is being done in this direction every day.^48–53^ In another study, asphalt binders in Malaysia were modified with nanoclay and bitumen additives (WAA), and test methods were used to determine their many properties. The results of the study showed that the modified bitumen composites remain stable during storage and improve the physical properties of asphalt concrete, such as increasing its softening temperature and reducing its brittleness temperature. In addition, the additives significantly reduce the mixing temperature and time of bitumen with a stone cover. Large-scale polymer waste is currently the biggest threat to the world and causes chemical pollution (Fig. 4). Extensive research is needed to develop various methods for the beneficial use of large amounts of polymer waste, and the solution to this is to use them as modifiers in bitumen and use them in road pavements. The road industry is the most optimal choice for the use of various polymer wastes. Various polymers have been shown to be potential modifiers to improve the strength and durability of asphalt, as they better resist various damages, such as thermal cracking, water damage and deformation.^54–59^ This study examined the technical aspects and highlighted the effects of several types of polymer additives in bitumen and their effects on both bitumen and asphalt. In addition, the review discusses the suitability, challenges and prospects of polymer-modified bitumen. This study investigates the effects of plastomer polymer additives on the physical, rheological and thermal properties of bitumen and provides a detailed comparison of their effects on the performance of asphalt-concrete mixtures. Studies have shown that adding different amounts of modifiers to bitumen improves various properties of asphalt; however, mixing and storage methods significantly affect the stability of the binder. In another article, polymer wastes were used as bitumen modifiers to impart flexibility, which led to delayed cracking (Table 7).

**Table 7:** Additives used to modify bitumen in ref. 56. Compiled by the authors based on the literature data

Bitumen	Modifier	Suitable addition	Method/test
60/70 penetration	EVA	5%	DSC, DSR, and fluorescent microscopy
B 40/50 penetration	Nitrile rubber	5%	DSC, DSR, TGA, and FTIR
B 100/130	PET	1–3%	SEM
B 60/70 penetration	Nano-clay	4%	Dynamic creep test
VG 40	Bakelite	1–5%	Penetration test

Previous studies have investigated the mechanism of the modifier's effect on the properties of bitumen in a bitumen-polymer mixture and at the same time studied the properties of polypropylene.^60–63^ The processes of melting and dispersing the polymer included in the bitumen composition, as well as the degree of its solubility in the bitumen composition as a composite, were investigated. The effect of polypropylene's molecular structure on the rheological, thermal and mechanical properties of the resulting composite was considered. The main stages of the polymer included in the bitumen composition as a composite were described, including the mechanism of thermal damage, homogenization, the determination of the optimal component ratio and mixing methods. An analysis of changes in the physicochemical properties of polymer-modified bitumen was presented, including the softening temperature, needle penetration depth, adhesion, and tensile length. The results of the study proved that the use of recycled polypropylene improves the heat resistance, brittleness and adhesion properties of bitumen. This allows its widespread use in asphalt pavements, significantly increasing their durability and resistance to climatic factors. In addition, various concentrations of polymer additives (from 4% to 8%) and their effect on structural changes in the bitumen binder were investigated. According to the data collected, the addition of polypropylene has a positive effect on the performance of bitumen, as it reduces plastic deformation and increases mechanical strength. The addition of recycled polypropylene as a bitumen modifier not only improves the quality of road surfaces, but also solves the problem of polymer waste disposal and protects the environment by reducing its negative impact on the environment. This opens up new prospects for further research in the field of polymer modification of bitumen and the development of more environmentally friendly and durable asphalt-concrete materials. In addition, not only recycled polypropylene, but also other polymers have been modified, which allows us to determine the optimal option for use in road construction. The combined use of structural polymer additives in bitumen for modified asphalt concrete improved road quality and increased the thermal stability of the base asphalt binder.^64–66^ ELTC is uniformly distributed, and the compatibility between the components of the modified asphalt binder is good, demonstrating the homogeneity of the modified asphalt binders. The results showed that all ELTC formulations improve the softening point and increase their resistance to plastic deformation in summer.

Studies have shown that increasing traffic flows, vehicle loading, and exposure to harsh weather conditions (rain, snow, cold, and heat) require more durable and stable asphalt pavements.^67–70^ Asphalt pavement failure can have a significant economic impact. The use of high-quality aggregates and polymer-modified bitumen is a key step in preventing these failures. Scientists reported using the reactive elastomer terpolymer Elvaloy (Elvaloy®), a common thermoelastic polymer, to modify bitumen with varying degrees of polymerization, using up to 2% bitumen in 0.5% increments. Physical and rheological tests were used to measure performance at high and intermediate temperatures. According to physical and test results, significant decrease and increase were observed due to the test methods. Rutting and fatigue resistance at high temperatures vary with increasing polymer incorporation rate. Overall, the studies conducted show that the additive has a positive effect on various properties of bitumen.

The road construction industry is interested in using alternative and sustainable binders to facilitate the production and installation of flexible pavements and enhance the performance of flexible pavements.^71–73^ The aim of another study was to evaluate the physical properties of bitumen mixtures synthesized with polyethylene (PE), ethylene vinyl acetate (EVA) and styrene-butadiene-styrene (SBS) as bitumen additives. The bitumen and polymers in the samples were heated to their melting temperatures; the bitumen was intensively mixed at 170–200 °C for 10–15 minutes. The degree of penetration of the polymer into the bitumen during the process was calculated based on the temperature, mixing speed, and so forth. The results of the penetration test showed that the polymers have a reinforcing effect on the bitumen, as evidenced by the decrease in the penetration values of the PE-modified bitumen from 51 to 57 decimeters for the bitumen mixed with SBS and from 58 to 3, 6 to 7, and 13 to 18 for the bitumen modified with EVA, respectively. The softening point of the polymer-added bitumen is positively affected; the softening point of PE-modified bitumen increases from 542 °C to 675 °C. Similarly, the softening point of bitumen with added styrene-butadiene-styrene increases from 57 °C to 72 °C for all polymer formulations, and the softening point of bitumen with added EVA increases from 57 °C to 61 °C. Polymer addition has a significant positive effect on the percentage of water resistance of bitumen. Bitumen with SBS-type polymer additives exhibits properties not typical of bitumen, such as elasticity, which characterizes the ability to undergo reversible elastic deformations. Elasticity at 250 °C determines the cross-linked structure of the modifier, which is maintained at low temperatures.^74–76^

The use of polymers such as SBR or LDPE affects the modification of rheological properties.^77–79^ Results of traditional asphalt binder tests showed that the addition of SBR or LDPE polymers increases the rigidity of the asphalt binder and reduces its temperature sensitivity. The polymers significantly improve the rheological properties of the asphalt binder, with the improvement increasing with increasing polymer content but varying depending on the polymer category. The addition of SBR and HDPE polymers to bitumen appears to have a positive effect on its softening temperature. With an increase in the SBR content from 3% to 9%, the softening point increases by approximately 27%. Increasing the LDPE content from 3% to 9% leads to an increase in the softening point from 54 to 61. Here, the addition of 8% low-density polyethylene has a significant positive effect on the softening temperature of the bitumen. In parallel, it was noted that the penetration properties of the bitumen in the low-density polymer asphalt mixture improve by about 10%. These results confirm that it is possible to produce high-quality asphalt by modifying traditional asphalt-concrete mixtures with granular polymer additives in the dry process.

The petroleum bitumen BND 90/130, produced at TOO SP Kaspiy Bitumen, was modified with a modifier containing a copolymer of ethylene with butyl acrylate and glyceryl methacrylate, taken in an amount of 0.5–1.6 wt%, as well as tire reclaim (4–20 wt%), which is a destructor of cross-linked elastomers of various chemical natures. The feasibility of using the developed bitumen-elastomer binders in road asphalt concrete has been scientifically substantiated.^80–82^ The modification of bitumen using an ethylene-butyl acrylate-glyceryl methacrylate copolymer has been reported to significantly enhance its performance characteristics. The modified binders exhibit higher softening points and hardness values, together with improved elasticity, low-temperature flexibility, and adhesion to metallic and mineral surfaces. These improvements were attributed to chemical interactions between the copolymer and the functional groups present in bitumen asphaltenes, primarily through the epoxy functionality of glyceryl methacrylate. Spectroscopic and compositional analyses further revealed an increase in the proportion of high-molecular-weight asphaltenes, accompanied by a slight rise in the content of structured resin fractions. Bitumen modified with rubber crumbs with a size of 0.6–1.0 mm has been found to be highly elastic. The most effective composition of a bitumen-reclaimed composite material based on tire reclaim was determined. Based on its combined physical, chemical, and operational characteristics, as well as its comparative cost, a bitumen-reclaimed composition with a reclaim content of 20% is the most suitable, surpassing the bitumen modified with an optimal ethylene content, butyl acrylate, and glyceryl methacrylate (1.6%) in terms of a range of properties. Cutting tires into small pieces and incorporating them into bitumen makes it both environmentally and economically viable. Rather than dumping such tires into the environment, it is more promising to use them as modifiers to improve the softening, elongation and adhesion properties of bitumen.

In recent years, replacing traditional polymer modifiers with plastic waste has attracted increasing interest.^83–85^ Implementing such a technology would significantly reduce both production costs and waste disposal volumes. Among other polymers, polyethylene has been shown to have the greatest effect on bitumen. The study presented in this article aims to investigate how polyethylene and waxes affect the softening temperature, adhesion and penetration depth of bitumen. The modifiers were mixed simultaneously in the bitumen composition. In fact, the presence of waxes reduces the polarity of the bitumen matrix and increases its affinity for the polymer, facilitating its dispersion. The results obtained show that the polyethylene mixture creates polarity in the bitumen composition by linking the chains. Short-chain waxes have a negative impact on rutting resistance, regardless of mixing conditions. Conversely, long-chain waxes improve the overall performance of polyethylene-modified binders, particularly their resistance to permanent deformation.

The physicochemical properties of bitumen constantly change, both during the production of asphalt concrete mixtures and during the service life of road surfaces. These additives cause the bitumen to harden, which leads to rapid cracking in asphalts made from this bitumen, which in turn leads to rapid failure of the asphalt. Therefore, the addition of polymers to protect asphalt from rapid aging and cracking has become the most relevant research topic from an economic point of view.^86–88^ In accordance with these trends, the main goal of this study is to slow down the rapid aging process by adding various chemical acids to bitumen. This article presented their effect on the aging process as well as on physicochemical and structural changes. Studies have shown that some acids, in particular chitosan, have the strongest antiaging properties. Its addition in the range of 1–3% serves to increase the softening temperature by 2–4 °C and reduce the dynamic viscosity by 10–20%. In addition, the use of the composite allows one to reduce the oxidation rate of bitumen components, which is confirmed in the production of asphalt. Bitumen can account for up to 60% of the total use in asphalt production.^89,90^ The main objective of another research work was to investigate the mechanism of damage to the physical and chemical properties of bitumen by acids as modifiers. A research work was carried out using a petroleum road bitumen sample from CASPI BITUM (Kazakhstan) and a PLA sample from Zhejiang Hisun (China). During the study, changes in the quality parameters of biopolymer bitumen were determined when 4–10% PLA was added. Recent studies have shown that biopolymers significantly affect the properties of bitumen. All biopolymer bitumen samples studied were found to exhibit increased plasticity at 25 °C (>100 cm). Adding 8% PLA to bitumen resulted in optimal-quality biopolymer bitumen. These results can be used in the production of biopolymer road bitumen.

The rheological properties, adhesion, and shear strength of modified rubber-bitumen binders were studied under various conditions. The study used 60/70 grade bitumen and added polymers to it. Rubber crumb was added to the modified bitumen based on its weight. Silane was used as a surfactant at a rate of 0.1% by weight of the asphalt binder. Rheological properties and recovery tests were conducted for all binders. To simulate crack movement in the pavement, adhesion tests and a parallel-layer direct shear (LPDS) test were conducted. The results showed that the addition of rubber crumb and latex positively affects the performance of the asphalt binder. Asphalt binders modified with rubber crumb and natural rubber latex have relatively identical performance.^91–95^

The rheological properties of bitumen with a penetration of 60–70, modified with styrene-butadiene rubber (SBR), an elastomer and a type of synthetic rubber, were studied. Styrene-butadiene rubber (SBR) is a copolymer mainly composed of styrene and butadiene units. Because bitumen exhibits viscoelastic behavior, its performance is highly dependent on both temperature and loading conditions. To evaluate the influence of SBR on binder characteristics, several properties, including complex shear modulus (G*), viscosity, penetration, softening point, and aging resistance, were investigated using standard techniques such as the dynamic shear rheometer (DSR), rolling thin-film oven test (RTFOT), pressure aging vessel (PAV), rotational viscometer (RV), and penetration tests. Bitumen binders containing different SBR concentrations (0–5 wt%) were examined. The incorporation of SBR significantly enhances the viscoelastic response of the binder, resulting in an increased complex shear modulus and improved resistance to permanent deformation. Furthermore, higher SBR contents are associated with increased binder viscosity, which consequently requires elevated mixing and compaction temperatures during asphalt mixture production.^96–98^

Asphalt pavements are extensively employed in the transportation infrastructure because of their durability and ability to sustain heavy traffic loads. In recent years, increasing attention has been directed toward the utilization of waste polymers as asphalt modifiers. This approach not only improves the mechanical and rheological performance of asphalt mixtures but also provides an environmentally sustainable solution for managing polymer waste. Consequently, the incorporation of recycled polymer materials into asphalt pavements has emerged as an effective strategy for enhancing pavement performance while reducing the environmental burden associated with plastic waste disposal.

Researchers have demonstrated that the incorporation of polymer waste into asphalt concrete mixtures can improve the performance of asphalt concrete pavements, particularly in preventing common damage, including permanent deformation, thermal cracking, and fatigue cracking.^99–108^ The aim of this process was to polymerize waste polymers into asphalt-concrete compounds, changing their properties by adding acids, as mentioned before, and to make environmentally friendly use of waste polymers.

In addition to academic studies, several industrial developments and patents have significantly contributed to the advancement of polymer-modified bitumen technologies (Table 8). Early patents developed by Kraton Polymers (EP1586606A1 and EP1612243A1) established the use of high-molecular-weight SBS block copolymers for improving the elasticity, storage stability, and rutting resistance of asphalt binders. More recently, industrial innovations have focused on alternative modifiers such as EVA-GMA terpolymer and recycled-plastic-based additives (*e.g.*, Eco Flakes technology), aiming to reduce production temperatures, improve sustainability, and decrease the carbon footprint of asphalt pavements. These developments demonstrate the growing transition from conventional SBS-modified binders toward more sustainable and economically viable polymer modification technologies.^108–112^

**Table 8:** Key features of major commercial and industrial patents on polymer-modified bitumen

Patent/technology	Modifier type	Main innovation	Main benefit
EP1586606A1	SBS	High-molecular-weight SBS modification	Improved elasticity and rutting resistance
SBS/LDPE patents	SBS + LDPE	Sulfur cross-linking and pre-blended polymers	Enhanced storage stability
EVA technologies	EVA	Plastomer modification	Increased stiffness and deformation resistance
Recycled plastic technologies	PE, PP, PET, and PVC	Waste-plastic utilization	Sustainability and improved rutting resistance
Eco-flakes technology	Recycled plastics	Direct asphalt modification	Reduced CO_2_ emissions and circular economy approach

## Conclusion and future perspectives

This review shows that the use of polymer modifiers in asphalt pavements plays an important role in improving the rheological, mechanical and durability properties of bitumen. The incorporation of various polymers, including elastomers, such as styrene-butadiene-styrene (SBS), styrene-butadiene rubber (SBR) and crumb rubber (CR), plastomers, such as polyethylene (PE), polypropylene (PP), polyethylene terephthalate (PET), and ethylene-vinyl acetate (EVA), and other reactive modifiers, significantly improves the performance and properties of bituminous binders and significantly improves the service life of road pavements. The reviewed studies show that polymer modification generally increases the softening point, viscosity, elasticity and resistance to permanent deformation while reducing temperature sensitivity and increasing crack resistance and significantly affecting the adhesion properties. Elastomeric modifiers, especially SBS, provide excellent elastic recovery and crack resistance, making them highly resistant to heavy vehicle loadings and extreme weather conditions (rain, snow, high and low temperatures). Plastomer modifiers, including recycled polyethylene and polypropylene, significantly improve strength and high-temperature performance, thereby reducing susceptibility to cracking and protecting against the environment. In addition, reactive modifiers and nanomaterials help improve the compatibility, storage stability and long-term performance of polymer-modified binders. A comparative analysis of the literature shows that the effect of the polymer modifier depends on its type, chemical composition, and solubility as well as the temperature. In many cases, the addition of 2–8 wt% polymer leads to significant improvements in rheological properties. Most studies have shown that low polymer contents can provide significant performance improvements while remaining economically viable. However, excessive polymer contents can negatively affect storage stability, workability and production costs. Therefore, optimization of the polymer dosage and processing conditions remains an important issue for practical applications, and future work should help to address this issue. The increased use of recycled polymers and industrial waste materials in bitumen modification offers significant environmental and economic benefits and meets the growing demand for sustainable pavement technologies. The use of waste plastics, crumb rubber and bio-based polymers not only improves the performance of pavements but also contributes to waste management and supports the principles of economy by reducing landfill disposal and the consumption of virgin raw materials. Despite significant advances in polymer-modified bitumen technology, a number of challenges remain. Compatibility between polymers and bitumen, phase separation during storage, long-term aging behavior and field performance under different climatic conditions still require further studies. In addition, standardized methodologies are needed to evaluate the rheological and durability properties of polymer-modified binders to facilitate comparisons between different studies.

Future research should focus on the following:

1. Development and industrial application of environmentally friendly and sustainable polymer modifiers based on recycled and bio-based materials;

2. Optimization of the polymer composition and dosage to achieve the best balance between low polymer addition and high quality along with high performance and low costs;

3. Enhancement of the compatibility and storage stability of polymer-modified binders with reactive additives and nanomaterials;

4. Investigation of the long-term field performance and aging mechanisms of modified binders under different traffic and climatic conditions;

5. Life cycle assessments and economic analysis to assess the sustainability of polymer-modified asphalt technologies;

6. Investigation of advanced characterization techniques and data-driven approaches, including artificial intelligence and machine learning, for the design and optimization of next-generation polymer-modified bitumen systems.

Overall, polymer modification has been proven as one of the most effective approaches to improve the rheological and performance properties of bitumen. Ongoing research and industrial development in this area are expected to lead to the production of more durable, sustainable, and environmentally friendly asphalt pavements that can meet the increasing demands of the modern transportation infrastructure.

## Conflicts of interest

There are no conflicts to declare.

## Data Availability

The data supporting the findings of this study are available within the article. Additional data supporting the findings of this study are available from the corresponding author upon reasonable request.
